# Total Fat Content and Fatty Acid Profile of Fine-Aroma Cocoa From Northeastern Peru

**DOI:** 10.3389/fnut.2021.677000

**Published:** 2021-07-05

**Authors:** Manuel Oliva-Cruz, Pati Llanina Mori-Culqui, Aline C. Caetano, Malluri Goñas, Nuri C. Vilca-Valqui, Segundo G. Chavez

**Affiliations:** ^1^Instituto de Investigación para el Desarrollo Sustentable de Ceja de Selva, Universidad Nacional Toribio Rodríguez de Mendoza de Amazonas, Chachapoyas, Peru; ^2^Facultad de Ingeniería y Ciencias Agrarias, Universidad Nacional Toribio Rodríguez de Mendoza de Amazonas, Chachapoyas, Peru

**Keywords:** cocoa butter, unsaturated fatty acids, saturated fatty acids, C18:0, C20:0, C16:0, C18:3, C18:1

## Abstract

Cocoa beans are the raw material for the chocolate industry. In this study, the total fat contents and fatty acid profiles of fine-aroma cocoa beans of 30 cocoa ecotypes from northeastern Peru were evaluated. Results showed that SJJ-1 and ACJ-11 ecotypes from San Martin and Amazonas regions, respectively, presented highest percentages of total fat with an average of 30.49%. With respect to fatty acid profiles, it was found that cocoa ecotypes are composed of 10 fatty acids (C14:0, C16:0, C16:1, C18:0, C17:0, C18:1, C18:2, C18:3, C20:0, and C22:0); based on this profile, 5 clusters were determined. Cluster 5 had the highest content of C17:0 fatty acid (0.47%); however, the clusters 1, 2, 3, and 4 had the lowest content of this fatty acid (0.37%, 0.32%, 0.32%, respectively). The clusters 3 and 4 showed the highest content of C16:0 fatty acid (31.13% y 28.97%, respectively). The clusters 3 and 5 contained the highest content of the acid C18:1 (27.08% y 26.82%, respectively). The PCA found that C18:0 and C20:0 fatty acids are correlated, and are fundamentally opposite to C18:1, C16:0, and C18:3 acids. These results may be useful in identifying raw material for the development of specialty chocolates with better nutritional value than traditional cocoa.

## Introduction

Cocoa, which belongs to the Malvaceae family and the genus *Theobroma* (1), is native to South America and is one of the most significant resources of Mesoamerica; in this region, of course, cocoa was domesticated and had relevant value in different trends such as ritual drink and money (2). Cocoa is currently an important commodity globally and the main ingredient in the manufacture of chocolate, whose value and quality are related to the unique and complex flavors of cocoa (3). For years, cocoa has been cataloged as the most economically important crop in the world, due to its fundamental role in the chocolate industry (4), and Peruvian cocoa beans and their byproducts are also highly valued worldwide, especially the *Criollo* variety as it is considered fine-aroma cocoa (5).

Currently, healthy eating is becoming increasingly important, and based on this trend, cocoa can be evaluated on its healthy attributes. This crop is considered a superfood due to its antioxidant capacity and polyphenol content, which are linked to potential health benefits (6), making cocoa the third most important provider of these compounds after fruits and vegetables (7). In addition, chocolate or cocoa can exert its effect by favorably altering the intestinal microbiota (8), as preclinical studies have shown that a cocoa-enriched diet modifies cell functions because it leads to a modulation of systemic and intestinal antibody synthesis (9).

On the other hand, it is worth mentioning that oils and fats in food are composed of four types of fatty acids: polyunsaturated, monounsaturated, saturated and trans fatty acids (10). In fact, cocoa butter is an outstanding by-product of the food industry (11). However, there are several factors, among them, geographical origin, climatic conditions, agronomic management, cultivar type and harvest index, that influence the physicochemical characteristics of total fats and the fatty acid profile of cocoa (12, 13). Further, Salinas & Bolivar (14) found that Venezuelan chocolates have high variability in their content of saturated, monounsaturated and polyunsaturated fatty acids, but trans fatty acids has opposite content, less than the limits established by the FAO, FDA and Food Standard Agency; and even the lowest in lipid-based chocolates which consist mainly in cocoa butter.

In Peru, despite the importance of cocoa beans, few studies have been carried out to determine the total fat content and the fatty acid profile of the butter of fine-aroma cocoa beans, given that these beans are so-called because of their special aromatic qualities (notes of fruit, flowers, herbs, caramel, nut, and wood). Therefore, the objective of this research was to evaluate the total fat content and the fatty acid profile of 30 ecotypes of fine-aroma cocoa from Amazonas, Cajamarca and San Martin regions, located in the north-eastern part of Peru.

## Materials and Methods

### Location of Origin

**Figure 1:**
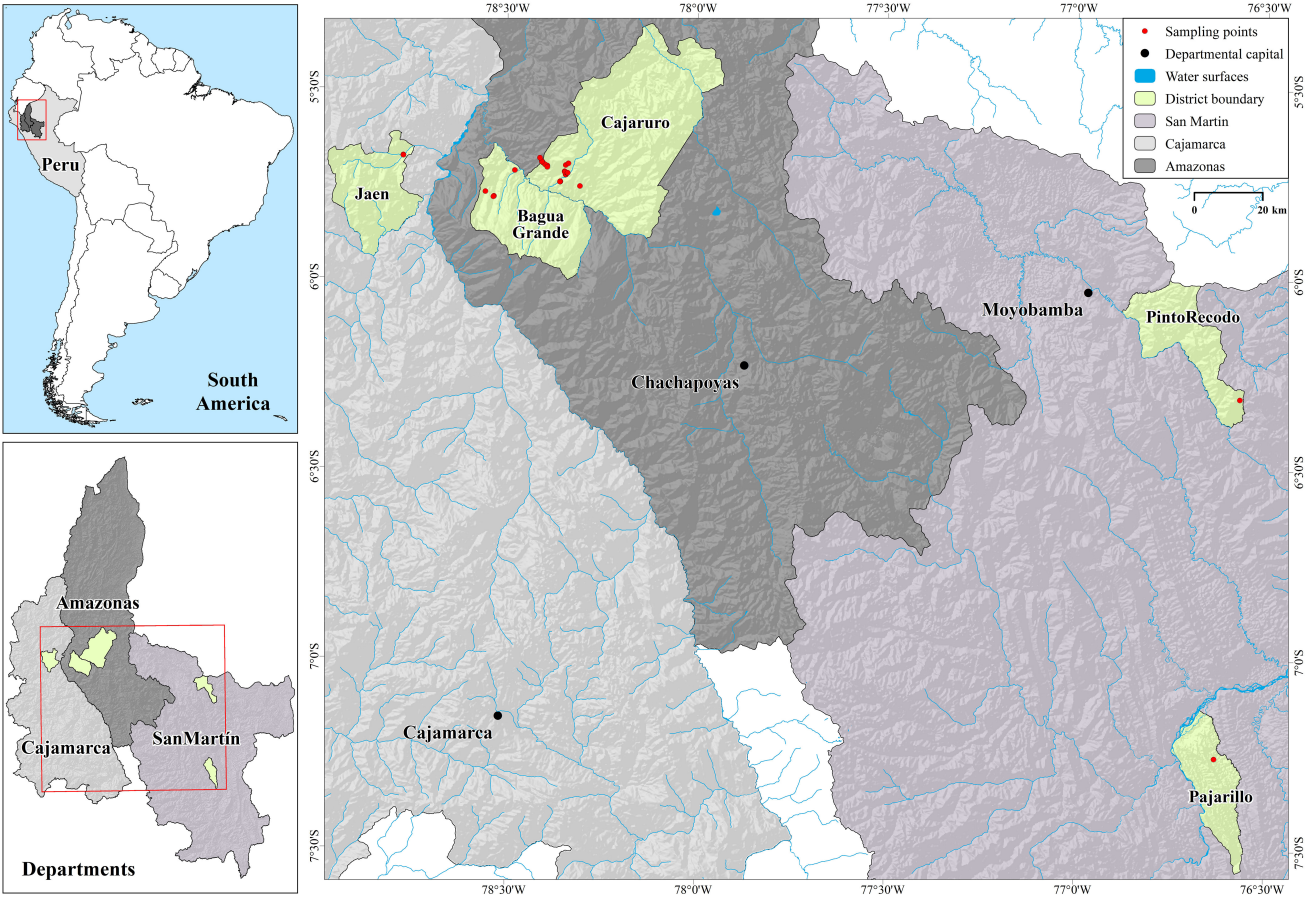
Location of origin of fine-aroma cocoa ecotypes.

The northeastern region of Peru, comprising Amazonas, Cajamarca and San Martin, is regarded as a highly important cocoa cultivation region. Hence, these three regions were selected as locations of origin for this study (Figure 1).

### Materials of Study

Thirty samples (500 g each one) of fermented and dried cocoa at 7.5% humidity, from different geographical origins were characterized: Amazonas (26 samples), Cajamarca (2 samples) and San Martin (2 samples). The samples were harvested between the months of April and September, 2018 by the Cocoa Research Circle (CINCACAO) technical service, of the Instituto de Investigación para el Desarrollo Sustentable de Ceja de Selva (INDES-CES) and sent to the Laboratorio de Investigación en Fiosología y Biotecnología Vegetal (FISIOBVEG), where the extraction and characterization analyses of the cocoa butter were carried out.

Each sample was assigned a code and a number. Each code was composed of representative letters of the name of the region of origin, district sector and the sequence in which it had been collected as follows: ecotypes of Amazonas, from Cajaruro1: ACJ-1 to Cajaruro 17: ACJ-17, Bagua Grande 1: ABG-1, Bagua Grande 2: ABG-2, Bagua Grande 3: ABG-3, Bagua Grande 4: ABG-4, Copallín 1: ACP-1, Copallín 2: ACP-2 and Copallín 3: ACP-3; ecotypes of the Cajamarca region Yanayacu 1: CYY-1 and Yanayacu 2: CYY-2; and ecotypes of San Martín, Juanjui 1: SJJ-1 and Pinto Recodo 1: SPR-1.

### Chemicals and Reagents

In order to extract total fats, we acquired petroleum ether from JT Baker, USA. To prepare fatty acid methyl esters (FAME), the following solutions were used: n-Hexane [purity; 99% (GC)] from Sigma-Aldrich, USA; sodium hydroxide (KOH) and hydrochloric acid (HCl) solution, from Merck, Peru; and methanol (purity; 99.8%) from Spectrum Chemical, USA. And finally, to quantify fatty acid methyl esters, we used Supelco standard, 37 Component FAME Mix [purity; ≥99% (GC)], from Sigma Aldrich, USA.

### Sample Preparation

From the general samples (500 g) of cocoa beans, a sample of 15 grams was randomly selected, the shells were removed manually and placed in a clean and coded polyethylene bag.

### Determination of Total Fat Content

Following the protocol for fat extraction of the Official Methods of Analysis (A.O.A.C.) (15) the extraction process was carried out with a semi-automatic soxhlet (J.P. Selecta F-6, Barcelona, España) containing petroleum ether (FP −49°C) as solvent. After optimization, the extraction time was adjusted to 3 h at 120 °C. Subsequently, the defatted cocoa nibs were discarded and the solvent-free butter was quantified and stored in amber vials at −20°C until further analysis.

### Preparation of Fatty Acid Methyl Esters (FAMEs)

Methylation, a previous stage to fatty acid identification on gas chromatograph, implies depolarizing carboxyl groups and converting them to FAMEs, which are more volatile than carboxyl groups and facilitate fat determination by gas chromatography with flame ionization detector (GC-FID) (16).

For each sample, methylation method proposed by Salimon et al. (17) was followed. Therefore, a total of 0.15 g of fat extract was placed (in triplicate) into a test tube with a screw cap (10 ml), and 2 ml of n-hexane with 1 ml of 2.0 M methanolic KOH solution were subsequently added to the samples. Afterwards, 1.2 ml of HCl (1.0 M) was added to the tubes and they were centrifuged at 4,032 G for 30 s and placed in a water bath for 2 min at 70 °C. After phase separation, 1 ml of n-hexane was added. The upper phase of the solution containing the FAME was transferred with a Pasteur pipette to an amber vial (2 ml), so as to do chromatographic analysis.

### Identification and Quantification of the Fatty Acids of Fine-Aroma Cocoa Ecotypes by GC-FID

The identification of FAME was carried out using an Agilent Technologies 7890B Series GC System (Santa Clara, California, U.S.A.), chromatograph with a flame ionization detector (FID). The separation was performed on a DB-WAX UI capillary column (30 m, I.D. 0.320 × 0.50 μm; Santa Clara, California, USA). One μl of sample was injected into a split mode (50:1) at a temperature of 250 °C. Helium was used as the carrier gas at a constant flow rate of 1.1 ml/min. The oven temperature programmed was: 1 min at 50 °C, 10 °C/min up to 200 °C, then 3 °C/min up to 230 °C, finally 20 min at 230 °C. The detector temperature was 280 °C. FAME analysis was performed by direct comparison of their retention times with a FAME standard. Quantification was performed by the external standard method, comparing the area existing in each sample in relation to the fraction contained in the FAME standard. All the analyses were conducted in triplicate.

### Statistical Analysis

The results of total fat content are presented as the mean values and standard deviations. In order to determine the differences between the total fat percentage of the cocoa ecotypes, analysis of variance was applied using the general linear and mixed models technique (GLMM) followed by the DGC multiple comparisons test (Di Rienzo, Guzman and Casanoves). To perform a numerical classification of the cocoa ecotypes, a hierarchical grouping of the fatty acid profiles was performed using the cluster analysis. Ward's algorithm and Mahalanobis distance were used to construct the tree, and then, a cut was made at 36.45% of the largest distance to establish the clusters. For each cluster, the average fatty acid profile was calculated. In addition, using the GLMM models technique followed by the DGC multiple comparisons test was used to determine the differences between the clusters. The principal component analysis (PCA) showed the correlation between the fatty acids analyzed. Finally, Pearson's correlation was applied to measure the relationship between the altitude of sample collection and the fatty acid profiles of the ecotypes studied. All data were analyzed using InfoStat/Professional software 2018p version.

## Results

### Total Fat

The total fat content in each of the 30 ecotypes studied were different, the analysis of variance and the multiple comparisons test separated the ecotypes into four groups and/or levels of total fat content, which were represented by a letter (a, b, c, and d) (Table 1). Group “a” grouped the ecotypes ACJ-11 and SJJ-1, which had high total fat contents (at 30.87 and 30.11%, respectively); group “b” grouped five ecotypes with total fat contents between 25.66 and 27.74%; however, group “c” grouped 9 ecotypes, and the total fat content was between 22.13 and 24.58%; finally, 14 ecotypes were in group “d”, and they had the lowest values of total fat content (from 17.5 to 20.82%).

**Table 1:** Total fat percentage of fine-aroma cocoa ecotypes (means ± standard deviation).

**Ecotype**	**Total fat (%)**	Meters above the sea^*^	**Ecotype**	**Total fat (%)**	Meters above the sea^*^
ACP-1	17.51 ± 3.52 d	597	ACJ-14	22.38 ± 1.38 c	665
ABG-4	17.63 ± 1.72 d	826	ACP-2	22.38 ± 0.39 c	432
ACJ-12	17.87 ± 0.54 d	703	ACJ-17	22.54 ± 1.08 c	803
SPR-1	18.05 ± 0.57 d	795	ACJ-6	23.03 ± 1.63 c	843
ACJ-5	19.25 ± 2.99 d	688	CYY-1	23.47 ± 1.58 c	737
ABG-3	19.64 ± 1.13 d	667	ACJ-10	23.75 ± 1.71 c	795
ABG-2	20.11 ± 1.34 d	666	ACP-3	24.31 ± 2.54 c	669
ACJ-3	20.22 ± 0.93 d	727	ABG-1	24.58 ± 0.70 c	727
CYY-2	20.22 ± 2.19 d	737	ACJ-4	25.66 ± 1.32 b	726
ACJ-15	20.26 ± 1.24 d	629	ACJ-16	26.23 ± 0.30 b	583
ACJ-19	20.42 ± 0.16 d	793	ACJ-18	27.50 ± 1.02 b	665
ACJ-2	20.56 ± 1.88 d	721	ACJ-9	27.59 ± 2.23 b	1,989
ACJ-7	20.70 ± 1.04 d	766	ACJ-13	27.74 ± 0.46 b	761
ACJ-1	20.82 ± 2.33 d	740	SJJ-1	30.11 ± 0.61 a	754
ACJ-8	22.13 ± 0.85 c	739	ACJ-11	30.87 ± 1.58 a	349

*Values expressed as the averages per 100 g of sample in the composition analysis of three samples. The values with different letters in a column are significantly different, according to the DGC test (P < 0.05)*.

* *Sample collection height*.

On the other hand, with respect to the geographical origin of the samples, 8 ecotypes from the Amazonas region were in group “c,” five were in group “b,” and one was in group “a.” Similarly, of the ecotypes from the Cajamarca region, one ecotype was in group “c,” and one ecotype was in group d. Finally, the ecotypes from the San Martin region were distributed in groups “a” and “d” (with one ecotype in each group).

Regarding the relationship between the total fat percentage of the studied ecotypes and the altitude above sea level showed in the Table 1, Pearson's correlation did not find a significant correlation (*p*-value = −0.11; Pearson = 0.30), which allows us to state that the total fat percentage of the ecotypes is independent of the sampling altitude.

### Identification and Quantification of Fatty Acids for the Classification of Fine-Aroma Cocoa Ecotypes

This study identified and quantified the 10 fatty acids present in the fine-aroma cocoa ecotypes from the northeastern zone of Peru, of which six were saturated fatty acids (C14:0, C16:0, C17:0, C18:0, C20:0, and C22:0), two were monounsaturated (C16:1 and C18:1), and two were polyunsaturated (C18:2 and C18:3).

Table 2 presents the fatty acid profile of 30 ecotypes of fine-aroma native cocoa. In general, the predominant fatty acids are C16:0, C18:0, and C18:1, which represent more than 80% of the fat composition of cocoa beans. On the other hand, it can be observed that the C22:0 was found in lower proportions and its presence was noted in only seven of 30 ecotypes studied, which suggests the low bioavailability of this fatty acid in cocoa beans.

**Table 2:** Fatty acid profile of 30 ecotypes of fine-aroma native cocoa from northeastern Peru (means ± standard deviation).

**Ecotype**	**C14:0 Myristic**	**C16:0 Palmitic**	**C16:1 Palmitoleic**	**C17:0 Margaric**	**C18:0 Stearic**	**C18:1Oleic**	**C18:2 Linoleic**	**C18:3 Linolenic**	**C20:0 Arachidic**	**C 22:0 Behenic**
ABG-1	0.11 ± 0.00	28.27 ± 0.21	0.42 ± 0.03	0.30 ± 0.02	41.49 ± 0.39	26.39 ± 0.64	1.70 ± 0.01	0.19 ± 0.03	1.14 ± 0.08	0.00 ± 0.00
ABG-2	0.13 ± 0.01	28.18 ± 0.03	0.42 ± 0.01	0.38 ± 0.01	41.72 ± 0.04	25.85 ± 0.04	1.86 ± 0.01	0.17 ± 0.01	1.31 ± 0.01	0.00 ± 0.00
ABG-3	0.12 ± 0.00	29.69 ± 0.05	0.44 ± 0.01	0.40 ± 0.00	39.92 ± 0.04	26.09 ± 0.01	1.71 ± 0.00	0.17 ± 0.01	1.27 ± 0.00	0.20 ± 0.01
ABG-4	0.00 ± 0.00	29.6 ± 0.12	0.46 ± 0.00	0.28 ± 0.00	40.56 ± 0.11	26.04 ± 0.06	1.63 ± 0.01	0.11 ± 0.05	1.31 ± 0.01	0.00 ± 0.00
ACJ-1	0.12 ± 0.02	27.54 ± 0.09	0.44 ± 0.01	0.52 ± 0.01	41.37 ± 0.22	26.95 ± 0.10	1.61 ± 0.01	0.17 ± 0.01	1.28 ± 0.02	0.00 ± 0.00
ACJ-10	0.11 ± 0.01	27.27 ± 0.11	0.41 ± 0.02	0.47 ± 0.04	41.42 ± 0.32	27.20 ± 0.19	1.76 ± 0.03	0.13 ± 0.03	1.24 ± 0.03	0.00 ± 0.00
ACJ-11	0.11 ± 0.01	26.99 ± 0.02	0.42 ± 0.01	0.38 ± 0.02	42.65 ± 0.04	26.34 ± 0.02	1.77 ± 0.01	0.15 ± 0.02	1.19 ± 0.01	0.00 ± 0.00
ACJ-12	0.06 ± 0.05	27.04 ± 0.26	0.36 ± 0.01	0.27 ± 0.01	43.10 ± 0.21	25.67 ± 0.06	1.70 ± 0.01	0.20 ± 0.05	1.44 ± 0.02	0.16 ± 0.14
ACJ-13	0.12 ± 0.01	26.73 ± 0.82	0.42 ± 0.06	0.31 ± 0.05	41.87 ± 1.34	27.40 ± 1.57	1.91 ± 0.11	0.14 ± 0.04	1.09 ± 0.06	0.00 ± 0.00
ACJ-14	0.04 ± 0.06	27.38 ± 0.07	0.39 ± 0.01	0.40 ± 0.01	42.53 ± 0.24	26.25 ± 0.06	1.63 ± 0.01	0.08 ± 0.03	1.31 ± 0.01	0.00 ± 0.00
ACJ-15	0.00 ± 0.00	30.09 ± 0.17	0.38 ± 0.01	0.34 ± 0.01	40.51 ± 0.18	25.61 ± 0.06	1.78 ± 0.01	0.05 ± 0.04	1.26 ± 0.01	0.00 ± 0.00
ACJ-16	0.00 ± 0.00	28.21 ± 0.44	0.36 ± 0.03	0.41 ± 0.02	43.84 ± 2.07	24.00 ± 1.70	1.74 ± 0.12	0.12 ± 0.05	1.32 ± 0.07	0.00 ± 0.00
ACJ-17	0.12 ± 0.00	28.43 ± 0.06	0.39 ± 0.00	0.33 ± 0.00	41.12 ± 0.05	26.41 ± 0.02	1.79 ± 0.00	0.19 ± 0.02	1.22 ± 0.00	0.00 ± 0.00
ACJ-18	0.00 ± 0.00	28.13 ± 0.06	0.43 ± 0.01	0.37 ± 0.01	42.50 ± 0.12	25.75 ± 0.09	1.39 ± 0.00	0.12 ± 0.07	1.30 ± 0.03	0.00 ± 0.00
ACJ-19	0.18 ± 0.01	27.44 ± 0.15	0.37 ± 0.01	0.43 ± 0.01	41.82 ± 0.20	26.21 ± 0.06	2.14 ± 0.02	0.17 ± 0.01	1.24 ± 0.01	0.00 ± 0.00
ACJ-2	0.06 ± 0.05	32.02 ± 5.01	0.40 ± 0.07	0.40 ± 0.07	40.05 ± 0.71	2,618 ± 0.43	2.75 ± 1.89	0.82 ± 1.15	1.00 ± 0.49	0.16 ± 0.14
ACJ-3	0.11 ± 0.01	28.46 ± 0.31	0.36 ± 0.01	0.43 ± 0.01	41.60 ± 0.33	26.16 ± 0.03	1.53 ± 0.02	0.12 ± 0.01	1.23 ± 0.02	0.00 ± 0.00
ACJ-4	0.12 ± 0.00	27.5 ± 0.11	0.38 ± 0.00	0.29 ± 0.01	41.80 ± 0.10	26.76 ± 0.03	1.61 ± 0.01	0.16 ± 0.00	1.25 ± 0.01	0.13 ± 0.12
ACJ-5	0.12 ± 0.00	27.54 ± 0.22	0.48 ± 0.00	0.32 ± 0.00	42.23 ± 0.20	25.82 ± 0.09	1.91 ± 0.02	0.16 ± 0.02	1.27 ± 0.01	0.14 ± 0.12
ACJ-6	0.11 ± 0.01	28.85 ± 0.21	0.00 ± 0.00	0.41 ± 0.05	39.76 ± 0.30	27.46 ± 0.16	1.50 ± 0.01	0.18 ± 0.01	1.19 ± 0.01	0.12 ± 0.11
ACJ-7	0.13 ± 0.00	28.22 ± 0.11	0.42 ± 0.01	0.36 ± 0.01	41.13 ± 0.14	26.68 ± 0.04	1.78 ± 0.00	0.16 ± 0.01	1.13 ± 0.01	0.00 ± 0.00
ACJ-8	0.03 ± 0.06	29.18 ± 0.04	0.37 ± 0.01	0.30 ± 0.01	41.27 ± 0.09	25.74 ± 0.01	1.78 ± 0.01	0.12 ± 0.01	1.21 ± 0.00	0.00 ± 0.00
ACJ-9	0.11 ± 0.01	27.34 ± 0.34	0.43 ± 0.00	0.52 ± 0.02	42.16 ± 0.52	26.32 ± 0.21	1.77 ± 0.02	0.15 ± 0.02	1.19 ± 0.02	0.00 ± 0.00
ACP-1	0.15 ± 0.02	28.70 ± 0.18	0.40 ± 0.02	0.29 ± 0.01	39.94 ± 0.20	27.31 ± 0.14	1.95 ± 0.04	0.13 ± 0.07	1.13 ± 0.00	0.00 ± 0.00
ACP-2	0.07 ± 0.06	28.34 ± 0.15	0.42 ± 0.01	0.43 ± 0.01	41.45 ± 0.18	26.28 ± 0.11	1.69 ± 0.01	0.12 ± 0.04	1.19 ± 0.04	0.00 ± 0.00
ACP-3	0.1 ± 0.01	30.36 ± 0.68	0.37 ± 0.00	0.34 ± 0.01	38.60 ± 0.37	27.27 ± 0.34	1.76 ± 0.06	0.12 ± 0.03	1.06 ± 0.00	0.00 ± 0.00
CYY-1	0.09 ± 0.00	27.73 ± 0.05	0.41 ± 0.01	0.24 ± 0.01	42.37 ± 0.03	25.77 ± 0.02	1.94 ± 0.01	0.15 ± 0.00	1.29 ± 0.01	0.00 ± 0.00
CYY-2	0.13 ± 0.01	28.19 ± 0.17	0.41 ± 0.01	0.26 ± 0.00	41.55 ± 0.12	26.16 ± 0.00	1.86 ± 0.02	0.14 ± 0.03	1.30 ± 0.01	0.00 ± 0.00
SJJ-1	0.11 ± 0.00	28.62 ± 0.09	0.38 ± 0.01	0.30 ± 0.01	40.48 ± 0.22	26.87 ± 0.12	1.63 ± 0.01	0.17 ± 0.01	1.23 ± 0.01	0.20 ± 0.00
SPR-1	0.00 ± 0.00	31.90 ± 0.07	0.39 ± 0.00	0.32 ± 0.01	37.66 ± 0.05	26.91 ± 0.02	1.70 ± 0.01	0.06 ± 0.02	1.05 ± 0.01	0.00 ± 0.00

**Figure 2:**
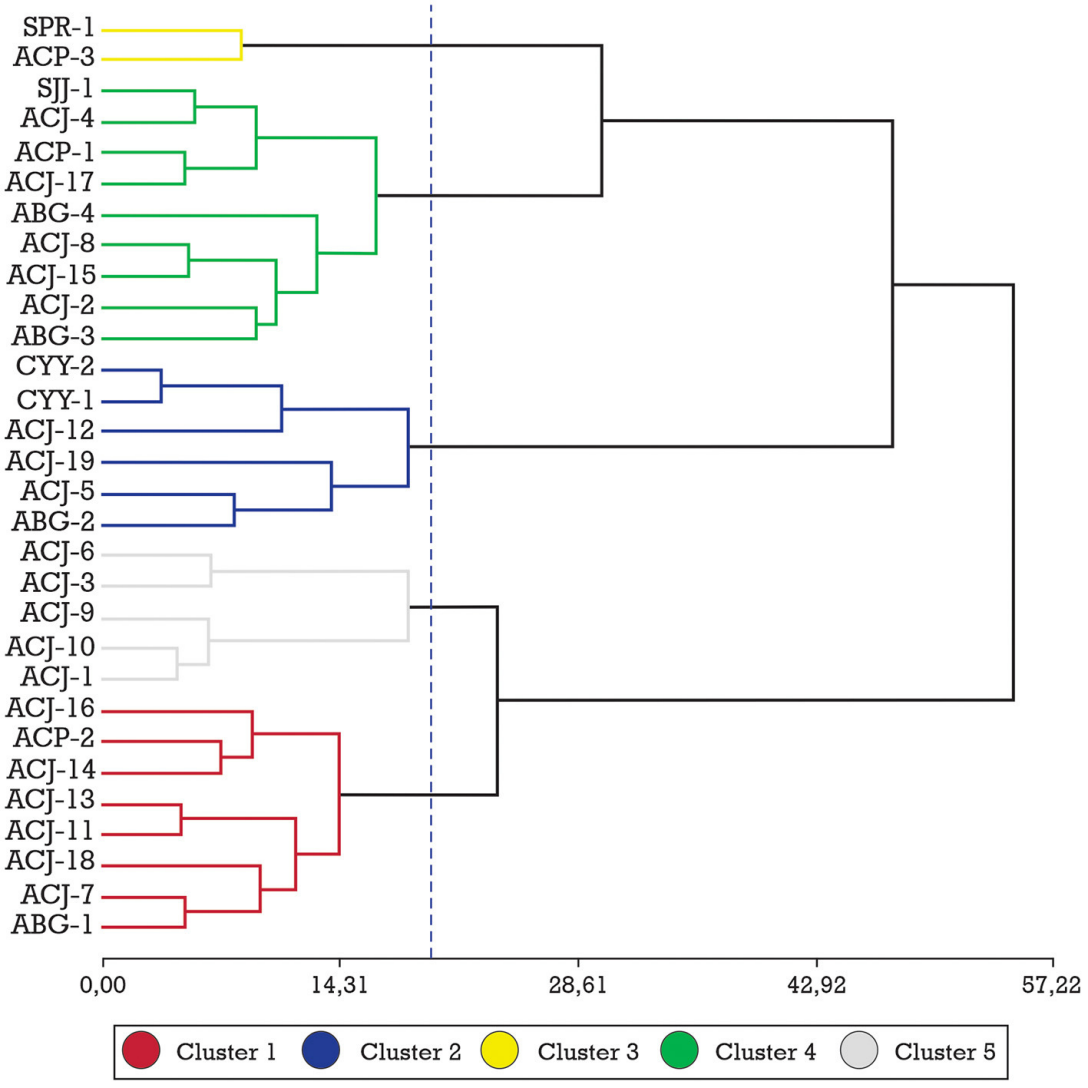
Ward method (20.0, 5 Groups) and Mahalanobis distance for 30 fine-aroma cocoa ecotypes from their fatty acid profiles.

After the cluster analysis, 5 clusters of fine-flavor cocoa were identified according to their fatty acid profiles (Figure 2). The dendrogram shows that cluster 1 is composed of 8 ecotypes from the Amazonas region; cluster 2 is composed of six ecotypes, four from Amazonas and two from the Cajamarca region; cluster 3 is composed of two ecotypes, one from Amazonas and one from San Martin; cluster 4 is composed of nine ecotypes, eight from Amazonas and one from San Martin; cluster 5 is composed of five ecotypes, all ecotypes from Amazonas region.

Table 3 shows the characteristics of the 5 clusters generated by their fatty acid profiles, and by performing the DGC mean comparison test; it was determined that the C22:0, C16:1, C18:2, and C18: 3 neither influenced nor determined the formation of these clusters, thus the concentration of these acids was not significantly different for any of the clusters formed, while the fatty acids C14:0, C16:0, C17:0, C18:0, C20:0, and C18:1, made a significant contribution to the formation of the clusters.

**Table 3:** Means of the fatty acid profile variables for the 5 clusters of 30 ecotypes of native fine-aroma cocoa from the northeastern zone of Peru.

**Lipid profile**	**Cluster 1**		**Cluster 2**		**Cluster 3**		**Cluster 4**		**Cluster 5**	
	***n* = 8**		***n* = 6**		***n* = 2**		***n* = 9**		***n* = 5**	
	**Mean**		**Mean**		**Mean**		**Mean**		**Mean**	
C14:0	00.07	b	00.12	a	00.05	b	00.08	b	00.11	a
C16:0	27.78	c	27.78	c	31.13	a	28.97	b	27.89	c
C17:0	00.37	b	00.32	c	00.33	c	00.32	c	00.47	a
C18:0	42.18	a	42.13	a	38.13	d	40.63	c	41.26	b
C20:0	01.21	b	01.31	a	01.06	c	01.21	b	01.23	b
C22:0	00.00	a	00.05	a	00.00	a	00.07	a	00.02	a
C16:1	00.41	a	00.41	a	00.38	a	00.39	a	00.41	a
C18:1	26.14	b	25.91	b	27.09	a	26.33	b	26.82	a
Grou	01.70	a	01.90	a	01.73	a	01.80	a	01.63	a
C18:3	00.13	a	00.17	a	00.09	a	00.19	a	00.15	a
Saturated	71.66		71.71		70.70		71.28		70.98	
Unsaturated	28.38		28.39		29.29		28.71		29.01	

*Variable means with different letters across rows are significantly different at P < 0.05*.

Furthermore, based on Table 4 resulting from the PCA and by taking the first four axes with a cumulative explained variance close to one, it explained over 50% of variance in the sample. In this context, clusters 2 and 5 had the highest concentration of C14:0 (0.12% y 0.11%, respectively) fatty acid compared to the other clusters; for C16:0 acid, clusters 3 and 4 had the highest concentrations (31.13% y 28.97%, respectively) in comparison to the clusters 1, 2, and 5, the same that had 27.78%, 27.78%, and 27.89%, respectively. For C17:0 acid, cluster 5 had the highest concentration (0.47%), followed by clusters 1, 2, 3, and 4 (0.37%, 0.32% y 0.33% y 0.32%, respectively), with lower concentrations of this acid.

**Table 4:** Auto-values of the principal component analysis.

**Lambda**	**Value**	**Proportion**	**Cumulative proportion**
1	2.67	0.27	0.27
2	2.01	0.2	0.47
3	1.67	0.17	0.64
4	1.19	0.12	0.75
5	0.9	0.09	0.84
6	0.78	0.08	0.92
7	0.45	0.04	0.97
8	0.17	0.02	0.98
9	0.16	0.02	1
10	3.20E−08	3.20E−09	1

Concerning C18:0 acid, clusters 1 and 2 had the highest concentration (42.18% y 42.13%, respectively) compared to clusters 3, 4, and 5 (38.13%, 40.63% y, 41.63%, respectively); while for C20:0 acid, cluster 2 had the highest concentration (1.31%), and cluster 3 had the lowest concentration (1.06%); finally for C18:1 fatty acid, clusters 3 and 5 had the highest concentration (27.09% y 26.82%, respectively), compared to clusters 1, 2, and 4 which had 26.14%, 42.13%, and 26.33% of this fatty acid, respectively.

As mentioned above, four principal components were selected, which together, explain 75% of the variability: 27% for the first axis, 20% for the second axis, 17% for the third axis and 12% for the last axis (Table 4). The correlation matrix of principal component (Table 5) shows that the first component depends on and correlates with C18:0 and C20:0 fatty acids, but C18:1, C16:0, and C18:3 are fundamentally opposite to this component. In the second component, the fatty acid with the highest negative correlation is C14:0, followed by C18:3 and C18:2, respectively, which in turn are opposite to C16:0. The third component depends on C22:0 and C17:0 which are negatively correlated and C14:0 and C18:0 which are positively correlated to this axis. Finally, the fourth component depends on C18:3 and C18:0 fatty acids.

**Table 5:** Correlation between originally measured variables and selected components.

**Variables**	**CP 1**	**CP 2**	**CP3**	**CP4**
C14:0 Myristic	−0.17	−0.71	0.46	0.09
C16:0 Palmitic	−0.59	0.73	−0.17	0.04
C17:0 Margaric	0.15	−0.17	0.56	0.31
C18:0 Stearic	0.88	−0.38	0.41	−0.43
C20:0 Arachidic	0.86	0.09	−0.15	−0.11
C22:0 Behenic	−0.16	−0.26	0.66	0.11
C16:1 Palmitoleic	0.15	−0.07	−0.44	−0.29
C18:1 Oleic	−0.62	−0.15	−0.49	0.06
C18:2 Linoleic	−0.38	−0.58	−0.04	0.32
C18:3 Linolenic	−0.42	−0.60	−0.25	0.83

*CP1, Component 1; CP2, Component 2; CP3, Component 3; CP4, Component 4*.

Pearson's correlation between sampling altitude and the fatty acid profiles of the study ecotypes were not significant, except for C17:0, which had a positive correlation, suggesting that C17:0 concentration levels increase at higher altitudes expressed in meters above sea level; contrary to C22:0 concentration as it decreases at the altitude increases (Table 6).

**Table 6:** Pearson's correlation of 30 ecotypes between the height of sample collection in meters above the sea level and the the fatty acid profile.

**Variable (1)**	**Variable (2)**	**Pearson**	***p*-value**
Altitud	C14:0 Myristic	0.12	0.2610
Altitud	C16:0 Palmitic	−0.13	0.2296
Altitud	C17:0 Margaric	0.25	0.0158
Altitud	C18:0 Stearic	0.02	0.8819
Altitud	C20:0 Arachidic	−0.08	0.4468
Altitud	C22:0 Behenic	−0.22	0.0335
Altitud	C16:1 Palmitoleic	0.19	0.0740
Altitud	C18:1 Oleic	0.13	0.2276
Altitud	C18:2 Linoleic	0.06	0.5996
Altitud	C18:3 Linolenic	0.03	0.7628

## Discussion

The average total fat contents of the ecotypes studied ranged from 17.51 to 30.87%. The formation of the four groups of ecotypes and their total fat content suggest that there are fine-aroma cocoa beans of high and low fat concentrations that may vary depending on the region of origin; however, the chemical compositions of cocoa beans also depend on the type of cocoa, degree of maturity, quality of fermentation and drying (12, 18). Similarly, genetic and environmental factors also play a very important role in the fat content and could explain why the ecotypes in this study had 38.87% as the highest value of total fat, which is below the value reported by Riaño H. et al. (13), at 53.76% total fat for cocoa from Peru. On the other hand, fine-aroma cocoa beans can have fat percentages below 50% (19), and by having low total fat contents, they have an advantage over hybrids, as they can have higher values of phosphorus, iron, zinc, sodium and selenium (20). However, the low concentration of total fat may be related to the size of the nibs particles used for fat extraction (21), and manual sample preparation may also be one of the causes of variation.

The identification of 10 fatty acids was consistent with those reported by MELO et al. (22), who found C14:0, C16:0, C17:0, C18:0, C18:1, C18:2, C18:3, C20:0, and C22:0 as the predominant fatty acids in chocolate. Comparably, C18:0, C18:1, and C16: 0 are the most representative fatty acids in descending order; but C16:0 and C18:0 are saturated fatty acids, which represent the largest fraction of fat in cocoa butter (23). This scenario explains the results obtained in the PCA, where C14:0, C16:0, C17:0, C18:0, C18:1, C18:3, C18:2, C20:0, and C22:0 were shown to be the most important, depending on the size of the vectors of PC1, PC2, PC3, and PC4. Similar results were reported by Torres-Moreno et al. (20), when studying the fatty acid profiles of different-origin cocoa, finding that quantitatively C16:0 (> 25%), C18:0 (> 33%), and C18:1 (> 34%) were the most important fatty acids in unroasted cocoa beans. The importance of C14:0 and C16:0 fatty acids expressed by PCA was because C14:0 acid is found in most vegetables and C16:0 is common in almost all organisms including many oils and fats (16). In parallel, when evaluating the fatty acid profiles of chocolates, palmitic acid (C16:0, 3.37–20.13 g/100 g), stearic acid (C18:0, 4.10–29.09 g/100 g) and oleic acid (C18:1, 4.17–27.08 g/100 g) are the most abundant in a sample (24). This suggests the dependence of the fatty acid profile of chocolate on the fatty acid profile of cocoa beans as raw material. Moreover, the determination of the fatty acids profile will help to choose cocoa beans taking into account the desired technological and nutritional characteristics (25).

Palmitic acid levels correlate negatively with stearic acid, oleic acid and linoleic acid (26). This same behavior was found in the PCA for palmitic acid, which was also correlated negatively with C18:0. Climate may be a factor for high levels of palmitic acid, which could be associated with the higher the temperature, the higher the concentration of this fatty acid (26).

The formation of the 5 clusters in the dendrogram was subject to the variability in the composition of the saturated and unsaturated fatty acids in cocoa, for many authors, the differences in the fatty acid profiles of cocoa are mainly explained by the effect of geographical origin (20). Fatty acid profiles are unique to cocoa and result in the characteristic texture and mouthfeel of the chocolate consumed (26). Based on this, cluster 3 was the group with the lowest percentage of saturated fatty acids (70.70%) and therefore the highest percentage of unsaturated fatty acids (29.29%), and cluster 2 was the ecotypes with the highest percentage of saturated fatty acids (71.71%) and the lowest percentage of unsaturated fatty acids (28.39%). It is stated that saturated fatty acids are harmful to human health if their consumption is excessive; however, there is a difference in C18:0 (present in greater quantity in clusters 1 and 2), compared to the rest of the saturated fatty acids; because studies based on humans have shown that it is a very poor substrate for the synthesis of triacylglycerols when compared to other saturated fatty acids such as myristic or palmitic, this shows that C18:0 has a protective factor against obesity (27).

On the other hand, polyunsaturated fatty acids cannot be synthesized by mammals, so they must be consumed as part of their diet (28, 29); among them are linolenic and linoleic acids, whose concentrations do not vary in any of the clusters. These acids produce energy, reconstitute the fatty tissue, and are found mostly in the seeds of vegetables and can meet 15 to 20% of daily energy needs (26). Therefore, according to McClements & Öztürk (29), omega-3 fatty acids, conjugated linoleic acids, carotenoids and phytosterols play an important role in the improvement of human health and well-being. These results highlight the importance of the representative concentrations of linolenic and linoleic acids in native fine-aroma cocoa beans from northeastern Peru. Based on Cabezas-Zabala et al. (30), the increased consumption of monosaturated and polyunsaturated fatty acids can reduce LDL cholesterol, resulting in a growing interest in incorporating these bioactive lipids in functional foods designed to produce certain health benefits, such as anti-inflammatory, antioxidant, anticancer and cholesterol-lowering properties (29). Cocoa protein and its peptides could be developed as an ingredient in the formulation of new functional foods aimed at mitigating obesity and associated disorders (31).

## Conclusions

The fine-aroma cocoa beans from northeastern Peru have a low percentage of total fat, which is influenced by the region of origin. The SJJ-1 and ACJ-11 ecotypes from the San Martin and Amazonas regions have the highest percentage of fatty acids, with an average of 30.49%. PCA demonstrated that C16:0, C18:0, C20:0, C18:1, C18:2, and C18:3 were the most important fatty acids in cocoa butter, depending on the size of the vectors. Cluster 3 grouped the ecotypes with the lowest percentage of saturated fatty acids (70.70%) and consequently, the highest percentage of unsaturated fatty acids (29.29%). Cluster 2 grouped the ecotypes with the highest percentage of saturated fatty acids (71.71%) and the lowest percentage of unsaturated fatty acids (28.39%).

The results of this research can be used by specialized cocoa buyers seeking raw material for the development of specialty chocolates with high nutritional value.

## Data Availability Statement

The raw data supporting the conclusions of this article will be made available by the authors, without undue reservation.

## Author Contributions

MO-C and SC: conceptualization. PM-C and NV-V: methodology. MG, NV-V, and MO-C: formal analysis. PM-C and AC: research and writing—preparing the original draft. MO-C, MG, and SC: writing—revision and editing. MO-C and SC: funding acquisition. All authors contributed to the article and approved the submitted version.

## Conflict of Interest

The authors declare that the research was conducted in the absence of any commercial or financial relationships that could be construed as a potential conflict of interest.
